# Catching the next wave? The relationship between UNESCO and developments in genomics

**DOI:** 10.1038/s41431-024-01621-y

**Published:** 2024-05-16

**Authors:** Oliver Feeney

**Affiliations:** Research Unit “Ethics of Genome Editing”, Institute of Ethics and History of Medicine, University of Tübingen, Tübingen, Germany

In 2025, the UNESCO/Universal Declaration on Bioethics and Human Rights will be 20 years old. At its 20th anniversary, it seems appropriate to ponder if there is justification for revising it, in particular, to improve and to clarify, to reflect new or previously underappreciated considerations, or to take account of any significant medical or technological developments that have occurred since its creation. One could say there is justification when one compares it to the 1964 World Medical Association’s Declaration of Helsinki (WMA [1]), which has undergone nine instances of amendments or clarifications* since its creation, with the intervals in revisions being, chronologically, eleven (1964–1975), eight (1975–1983), six (1983–1989), seven (1989–1996), four (1996–2000), two (2000–2002)*, two (2002–2004)*, four (2004–2008), and five (2008–2013). With the forthcoming revision expected later this year, the last interval will again be eleven years.

Of course, one could reply that it would be unfair to make *this* comparison for, in some important respects, it would be more accurate to compare a singular, revisable Declaration of Helsinki with a range of UNESCO declarations, and associated reports, which – when taken together – make for a form of revisable ‘family of declarations’ – albeit where each ‘revision’ is a new declaration. This can be seen clearly in terms of the arguably most significant medical-technological developments in the last decades, namely developments in human genomics. Gaydarsky and colleagues [2] capture this nicely with a very interesting and timely paper that seeks to illustrate how changes in the development of genetic technology in the last 20–30 years is reflected in the succession of UNESCO declarations, namely the 1997 Universal Declaration on the Human Genome and Human Rights (UNESCO [3]), the 2003 International Declaration on Human Genetic Data (UNESCO [4]), the aforementioned 2005 Universal Declaration on Bioethics and Human Rights (UNESCO [5]), and the 2015 Report of the International Bioethics Committee (UNESCO [6]). Their paper is an important overview of how such international declarations can be seen as products of their time and can be examined to indirectly see the state of the scientific realities at the time of their drafting, as well as contemporaneous societal, legal, and ethical priorities. There are three waves, as the authors call them, relating to the evolving focus of the declarations plus report, where one can view each wave as illustrating different ethical values being prioritised due to the specific ethical challenges raised by contemporaneous developments in the technology. From the initial prioritisation on the view of the human genome as a collective heritage of a unified humanity, the focus seemed a fundamental one aiming to balance scientific progress with notions of inherent dignity, alongside notions of autonomy, equality, and solidarity. With the second wave, we can see a greater specificity of such broad ethical concepts toward a more practical guidance on the use of human genetic data, and addressing issues such as consent, privacy, and non-discrimination. From the general ‘humanity focus’ of the first wave to the more individual focus of the second wave, with the third, we turn to a broader ethical outlook beyond individual rights, considering societal responsibilities, social discrimination, and group vulnerabilities. Throughout, we can see how long-standing values do not fundamentally change due to the evolution of genomic technology over the last 30 years – from the Human Genome Project to genome editing in the post-CRISPR Revolution era. However, which values are *prioritised* may change, and may manifest themselves in different guises. For instance, the generation of genetic data gives rise to challenges over who consents and controls to their data being recorded and stored; new forms of somatic gene therapies on improving hearing in deaf, or hard-of-hearing, infants, gives rise to ethical challenges over the distinctions between disease, disability, or diverse forms of the human good (Parens and Johnston [7]).

Returning to the initial reflection on the 20th Anniversary of the 2005 UNBHR, one might wonder what the next UNESCO revision may entail – meaning what may be in a future declaration or report with regards to ongoing developments in genome editing, including issues of access and equity to newly approved CRISPR therapies (such as can be seen with the costs of Casgevy and the socio-economic disadvantaged position of the majority of sickle-cell disease sufferers globally). Not only genome editing, but developments in polygenic screening, AI in genomics, and social and behavioural genomics may influence the content of future declarations or reports, and shape the prioritisation of what values are to be safeguarded.

Whether a new revision (i.e. new declaration) will be necessary and called for, due to the above emerging challenges, depends on a number of factors. Firstly, the authors note some of these challenges and have recourse to the already existing declarations and report. Whether or not this is sufficient, one can concede that such issues may still reside within the parameters of their ‘third wave’. One can perhaps see a lesson from the developments in CRISPR and Article 13 of the 1997 Council of Europe’s Oviedo Convention, where the Steering Committee for Human Rights in the fields of Biomedicine and Health (CDBIO [8]) re-examined the 1997 Convention to see if changes were needed to reflect advances in genome editing technologies in recent years. Only slight minimal changes in the form of clarifications were deemed necessary. Secondly, this connects to a wider issue on the degree of detail and fine-tuned guidance such declarations should contain for meeting various technological and medical developments. As technology evolves and grows in complexity, the question is whether declarations should also grow in complexity, to seek to address each and every new ethical issue that may arise. Or if they should restrain themselves to safeguarding a small number of general, universal, and unchanging ethical principles that would be applicable to all scenarios, but without attempting more fine-tuned guidance itself. With regard to the Declaration of Helsinki, Ehni and Wiesing [9] report differing views regarding whether it should be a highly detailed living document, adapting to rapid developments in medical research, or if it should consist of a short list of unchanging ethical principles that apply to every scenario, rapidly evolving or not. Gaydarska et al.’s [2] preference here may slightly tilt toward the short list, when they note that “achieving international consensus requires that at last the core elements of the declarations or report should not be easily changed over time, regardless of the changes in technology and society”. However, this would not rule out the more detailed, complex route either – as long as this greater fine-tuning worked within the parameters of a short number of constant and unchanging values and principles. Gaydarska et al.’s [2] overview also suggests that, even with a core of unchanging values or principles, there can be substantially different ethical priorities, or balancing of values, entailed depending on the technology and its application. Whatever new scientific developments may arise in the future, Gaydarsky et al.’s [2] paper will be a valuable guide to help understand and anticipate what new regulatory developments may arise in response.
